# Lacrimal gland enlargement in inactive thyroid eye disease: a case series and literature review

**DOI:** 10.5935/0004-2749.2024-0113

**Published:** 2024-09-16

**Authors:** Abdullah M. Khan, Abeer Alawi, Sahar M. Elkhamary, Osama Sheikh, Hamad M. Alsulaiman, Rajiv Khandekar, Silvana A. Schellini

**Affiliations:** 1 Oculoplastic Division, King Khaled Eye Specialist Hospital, Riyadh, Saudi Arabia; 2 Radiology Department, Mansoura Faculty of Medicine, Mansoura, Egypt; 3 Department of Ophthalmology, Faculty of Medicine, University of British Columbia, Vancouver, Canada; 4 Department of Ophthalmology, Faculdade de Medicina, Universidade Estadual de São Paulo “Júlio de Mesquita Filho”, Botucatu, SP, Brazil

**Keywords:** Graves’ ophthalmology, Graves’ disease, Lacrimal apparatus, Lacrimal apparatus diseases, X-ray computed tomography

## Abstract

**Purpose:**

This study aimed to evaluate the morphometric and volumetric dimensions of the lacrimal gland in patients with inactive thyroid eye disease and compare them with the values reported in the literature.

**Methods:**

This case series evaluated consecutive patients with inactive thyroid eye disease treated at a tertiary eye hospital from 2015 to 2020. The patients’ baseline demographics and clinical characteristics were obtained. The axial and coronal length, width, and volume of the lacrimal gland were measured on computed tomography scan images, and the results were statistically analyzed.

**Results:**

A total of 21 patients (42 orbits) with inactive thyroid eye disease were evaluated. Their mean age was 49.0 ± 14.6 years, and 12 (57.1%) of them were men. The main complaint was dryness, and the majority of the patients had good vision and mild proptosis. The mean axial length and width of the lacrimal gland were 19.3 ± 3.9 mm and 7.5 ± 2.1 mm, respectively; coronal length and width, 20.4 ± 4.5 mm and 7.5 ± 2.1 mm, respectively; and lacrimal gland volume, 0.825 ± 0.326 mm^3^. Age, sex, or laterality were not found to be determinants of lacrimal gland enlargement.

**Conclusion:**

Patients with thyroid eye disease have enlarged lacrimal gland even in the nonactive phase of the disease multifactorial aspects influence the lacrimal gland in thyroid eye disease, making it difficult to establish a clear correlation with predisposing factors. Further studies are warranted to better understand the association between thyroid eye disease and the lacrimal gland.

## INTRODUCTION

Thyroid eye disease (TED) is an autoimmune disease that induces an inflammatory reaction in orbital and adnexal tissues, which results in several changes to the oculo-palpebral region, including proptosis, lagophthalmos, lid retraction, extraocular muscle (EOM) enlargement, orbital fat infiltration, ocular surface abnormalities, and lacrimal gland (LG) changes^(^1^,^2^)^.

The LG is a target tissue, particularly in autoimmune and granulomatous diseases. Approximately 65%-85% of patients with TED^(^3^)^ exhibit enlarged LG, which can be the initial manifestation of the disease^(^4^)^. Hence, the evaluation of LG dimensions enables early diagnosis and timely treatment of TED^(^5^)^.

LG enlargement is a characteristic finding during the active phase of TED^(^1^,^3^,^5^,^6^,^7^,^8^,^9^)^. However, quantitative image studies on LG in TED are scarce, and there is a lack of reports on LG dimension during the inactive phase of the disease.

In this case series, we measured the LG dimensions via computed tomography (CT) in patients with inactive TED and compared the measurements with those in the literature to expand knowledge in this field.

## METHODS

This retrospective case series evaluated patients with inactive TED treated in a tertiary hospital from 2015 to 2020. The study protocol was approved by our Institutional Ethics Committee (1729-R), and the requirement of informed consent was waived due to the retrospective nature of the study.

**Inclusion criteria:** All consecutive TED patients with clinical activity score (CAS) <3 according to Group on Graves’ Orbitopathy diagnostic criteria^(^10^)^, with no prior history of immunosuppressive therapy or retrobulbar radiotherapy, and with a complete orbital CT scan data available for assessment.

**Exclusion criteria:** Patients with active TED (CAS ≥3) or other disease involving the orbital structures regardless of the etiology.

The baseline background of the patients was evaluated in terms of demographics, disease history, comprehensive ocular examination (eyelid position, EOM dysfunction, and orbital parameters-exophthalmometry, LG enlargement), and laboratory exams (level of T3/T4, thyroid-stimulating hormone (TSH), specific antibodies related to TED).

Proptosis was based on image examination and considered to be a dichotomy variable (yes/no). EOM dysfunction was classified as mild, moderate, or severe.

### Image examination and processing

**Figure 1:**
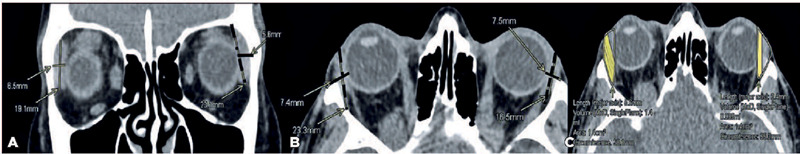
Noncontrast computed tomography (CT) in the axial and coronal planes of the lacrimal gland and orbit of patients with thyroid eye disease (TED). (A) Axial length and width in the axial CT image. (B) Coronal length and width in the coronal CT image. (C) The lacrimal gland is outlined with a pencil tool in the axial image.

CT scan images were reviewed on a picture archiving and communication system workstation using a commercial AGFA Enterprise Imaging workstation. The CT scan data were acquired using the Discovery 750 HD 64 Slice scanner (GE Healthcare, Milwaukee, WI, USA), with a scanning protocol of 0.6-mm axial, nonoverlapping contiguous sections for the orbits. This was achieved by tilting the patient’s head parallel to the Frankfurt plane. Quantitative measurements of the LG were performed by a senior neuroradiologist (SE). Each orbit of the patient was considered as one unit for calculation. Two measurements of both LG parameters were performed on the selected images using a previously described method^(^11^,^12^)^. The axial and coronal soft tissue series were the places where the LG appeared the largest to document dimensions in millimeters. The axial length (AL) of the LG was defined as the distance between the most anterior tip to the most posterior tip of the LG. Axial width (AW) was measured from the lateral to the medial edge at its widest point perpendicular to the length line on the axial images (Figure 1A). Coronal length was measured from the superior to the inferior tip of the LG. Coronal width (CW) was measured from the lateral edge to the medial edge at its widest point perpendicular to the length line on the coronal images (Figure 1B). The LG volume was obtained using a manual tracer approach following the delineation of the LG borders in each section on the axial and coronal images; the results were reported in cubic centimeters (Figure 1C).

### Statistical analysis

Data were collected from the patients’ electronic medical records in an Excel spreadsheet (Microsoft Corp., Redmond, WA, USA) and then transferred to a Statistical Package for Social Sciences spreadsheet (SPSS-23; IBM Corp., Armonk, NY, USA). The results of the clinical, laboratory, and imaging examinations were analyzed by two authors to evaluate the possible association between the variables. Descriptive analysis was employed to report clinical data and LG dimensions. P<0.05 was considered to indicate statistical significance.

## RESULTS

### Baseline demographic and clinical characteristics

The study group consisted of 21 patients with TED (42 orbits), with a mean age of 49.0 ± 14.6 years. Of them, 12 (57.1%) were male, 16 (76.2%) were nonsmokers, and 18 (85.7%) had other systemic health issues. Furthermore, 9 (21.4%) patients had glaucoma and 13 (61.9%) previously underwent orbital decompression surgery. The T3 hormone was within the normal range in 17 (81%) patients, the T4 hormone in 13 (61.9%), and the TSH hormone in 6 (28.6%). The main ophthalmic complaints were related to dryness (foreign body sensation, tearing, and redness) or EOM dysfunction (diplopia, gaze limitation). Only 4 (19%) patients complained of LG enlargement. The best corrected visual acuity was >20/50 in 27 (64.3%) eyes. Furthermore, 10 (23.8%) eyes exhibited lagophthalmos, 20 (47.6%) had mild proptosis, 12 (28.6%) had normal EOMs, and 11 (26.2%) presented with orbital apex crowding.

### LG dimensions in inactive TED patients

The LG in the 42 orbits of the 21 inactive TED patients had a mean AL of 19.3 ± 3.9 mm, mean AW of 7.5 ± 2.1 mm, CL of 20.4 ± 4.5 mm, CW of 7.5 ± 2.1 mm, and a volume of 0.825 ± 0.326 mm^(^3^)^. The LG dimensions of our patients in conjunction with other studies from the literature review involving normal subjects and TED patients are shown in table 1.

**Table 1:** Dimensions of the lacrimal gland in published papers on thyroid eye disease added to our series of cases

Authors	CT scan normal values					CT scan TOD values				
	Axial length	Axial width	Coronal length	Coronal width	Volume	Axial length	Axial width	Coronal length	Coronal width	Volume
Tamboli et al., 2011^(^11^)^	14.7 ± 19	5.1 ± 1.6	17.7 ± 3	5.2±1.6	-					-
Harris et al., 2012^(^13^)^						*20.1 ± 3.2 **19.7 ± 3.6	6.6 ± 2.0 6.4 ± 2.3	21.0 ± 3.7 20.7 ± 3.6	6.5 ± 2.0 7.1 ± 1.8	
Lee et al., 2013^(^14^)^	14.9 ± 2.2	4.1 ± 0.8	20.9 ± 2.7	3.6±0.7	-					-
Bingham et al., 2014^(^6^)^					0.696 ± 0.26					0.890 ± 0.35
Bukkari et al., 2014^(^15^)^					0.770 ± 0.34					
Hyun et al., 2014^(^16^)^					0.655 ± 0.15					
Huh et al., 2016^(^1^)^					0.608 ± 0.04					0.816 ± 0.05
Hu et al., 2016γ^(^5^)^	14.0 ± 1.9	3.8 ± 0.8	15.3 ± 2.7	4.3 ± 0.7	0.445 ± 0.19	16.2 ± 3,5	5.7 ± 3.1	16.2 ± 3.5	5.4 ± 1.0	0.713 ± 0.30
Danjen & Salaan, 2016^(^7^)^	14.6	4.1	20.7	2.9	-					-
Bubul et al., 2016^(^17^)^	16.2 ± 2.00	4.1 ± 0.7	18.3 ± 2.3	4.1 ± 0.7	0.617 ± 0.21					
Nawaz et al., 2020^(^18^)^	13.5 ± 1.8	4.2 ± 0.81	15.46 ± 1.96	3.99 ± 0.8	-					-
Starcevic et al., 2023^(^19^)^	*14.4 ± 2.6*14.9 ± 2.7	4.4 ± 1.04.6 ± 1.2	14.6 ± 2.314.9 ± 2.1	4.1 ± 1.14.1 ± 00.9	0.499 ± 0.1960.521 ± 0.25	*17.8 ± 3.5*16.9 ± 3.0	5.8 ± 1.95.6 ± 1.2	17.8 ± 2.916.9 ± 3.1	4.9 ± 1.55.1 ± 1.1	0.763 ± 0.450.735 ± 0.25
Present study						19.3 ± 3.9	7.5 ± 2.1	20.4 ± 4.5	7.5 ± 2.1	0.825 ± 0.33

* OD= male; **OD= female; *γ* = MRI evaluation discriminating active and inactive cases.

### Correlation between LG volume and demographic data

Our results indicated that sex, laterality, and age are not determinants of LG volume in TED (Table 2).

**Table 2:** Lacrimal gland volume in patients with thyroid eye disease and its determinants

		Mean	SDV	Validation
Sex	Male	0.85	0.36	Diff of mean 0.05 (95% Cl -0.16; 0.26, p=0.6
	Female	0.79	0.29	
Eye	Right	0.83	0.33	P = 0.97
	Left	0.82	0.33	
Age				R=-0.026, p=0.88
Lagophthalmos	Yes	1.04	0.43	Diff of mean -0.27 (95% Cl -0.63; 0.09, p = 0.12
	No	0.77	0.28	

## DISCUSSION

Our study demonstrated that the AL, AW, CL, CW, and volume of the LG were large in patients with inactive TED, consistent with the findings of the literature^(^1^,^5^,^6^,^13^,^19^)^.

The LG of our patients was measured on CT scan images in the same way as the others^(^1^,^6^,^13^,^19^)^. However, LG quantitative assessment via magnetic resonance imaging (MRI) has revealed that patients with TED have notably enlarged LG than normal individuals^(^1^,^3^,^5^,^6^,^7^,^11^,^14^,^15^,^16^,^17^,^18^,^19^,^20^)^. Although CT scan can be employed to assess LG, MRI exhibits higher sensitivity for the detection of active inflammation in TED^(^14^)^.

LG enlargement was previously associated with inflammatory TED activity^(^4^,^6^,^18^)^. However, only one study has differentiated the inactive and active phases of the disease^(^5^)^ being the other not homogeneous, including patients regardless of disease stage^(^1^,^6^,^13^,^19^)^.

Although LG prolapse appears to be a good indicator of disease activity in TED^(^3^)^, our results indicated that the LG remains enlarged even in the inactive phase of the disease, probably due to the remnants of fibrosis, glycosaminoglycan deposition, fatty infiltrations, and persistent interstitial edema^(^5^,^21^)^.

Our results also indicated that sex, age, and laterality are not determinants of LG enlargement. However, LG can be reduced in the elderly, with no sex variations^(^1^)^.

We measured the LG of both eyes but did not compare the laterality dimensions. This is a controversial issue, with some authors reporting asymmetric increase in the LG^(^11^,^17^,^22^)^ and others stating that no asymmetry exists between both sides in normal subjects or TED patients^(^1^,^6^,^7^,^12^)^.

In the present study, we did not compare the LG volumes in current smokers. However, smokers may have enlarged LG^(^1^,^13^)^.

Furthermore, we did not analyze the EOM dimensions in our patients. EOM enlargement occurs in conjunction with the progressive enlargement of the LG and proptosis in patients with TED^(^3^)^.

Our patients mainly presented with mild proptosis. Proptosis in patients with TED can be asymmetric, bilateral, or unilateral as well as mild or severe. The increase in LG volume is positively correlated with proptosis^(^14^)^ as the congestion of the retrobulbar orbital tissues in the active disease can push the orbital and LG forward, leading to LG prolapse and orbital tissue proptosis^(^3^)^. However, we did not evaluate this parameter as the majority of our patients had mild proptosis and were not in the active phase of the disease.

This study has several limitations. First, the sample size was relatively small, compromising correlation analysis with other parameters. Second, we used CT scans to analyze the dimensions and volume of the LG in patients with inactive TED. Although CT scan is considered to be a diagnostic tool for TED in clinical practice and for monitoring disease severity or progression^(^19^)^, MRI with diffusion-weighted imaging and the apparent diffusion coefficient of LG can better differentiate patients with active and inactive TED from healthy controls^(^5^,^9^,^20^)^. The recognized parameters correlated with increased LG dimensions include CAS, proptosis, and EOM volume^(^22^)^. However, only inactive patients with mild proptosis were studied, and the EOM volume was not evaluated, thereby compromising further analyses.

Nevertheless, our findings provide a basis for future studies. A larger sample size and a cohort of patients who are in other stages of the disease are warranted to better correlate the anatomical measurements of the LG with functional or pathological TED assessment. This would elucidate the association between LG and TED pathogenesis.

The LG is enlarged in patients with TED, even in those who are in the nonactive phase of the disease. However, due to the multifactorial aspects influencing the LG in TED, it was impossible to address the correlation with predisposing factors. Further studies are warranted to better understand the association between LG and TED.
